# Design Strategies for Biocontainment Units to Reduce Risk During Doffing of High-level Personal Protective Equipment

**DOI:** 10.1093/cid/ciz617

**Published:** 2019-09-13

**Authors:** Maria F Wong, Zorana Matić, Gabrielle C Campiglia, Craig M Zimring, Joel M Mumma, Colleen S Kraft, Lisa M Casanova, Francis T Durso, Victoria L Walsh, Puja Y Shah, Andi L Shane, Jesse T Jacob, Jennifer R Dubose

**Affiliations:** 1 SimTigrate Design Lab, Georgia State University, and College of Design; 2 School of Psychology, Georgia State University, and Georgia Institute of Technology; 3 Division of Infectious Diseases, Georgia State University, and Department of Medicine; 4 Department of Pathology and Laboratory Medicine, Georgia State University, and Emory University School of Medicine; 5 School of Public Health, Georgia State University; 6 Division of Pediatric Infectious Diseases, Department of Pediatrics, Emory University School of Medicine, and Children’s Healthcare of Atlanta, Georgia

**Keywords:** Ebola, occupational health, biocontainment unit design, design improvements, doffing personal protective equipment

## Abstract

**Background:**

Few data exist to guide the physical design of biocontainment units, particularly the doffing area. This can impact the contamination risk of healthcare workers (HCWs) during doffing of personal protective equipment (PPE).

**Methods:**

In phase I of our study, we analyzed simulations of a standard patient care task with 56 trained HCWs focusing on doffing of high-level PPE. In phase II, using a rapid cycle improvement approach, we tested different balance aids and redesigned doffing area layouts with 38 students. In phase III, we tested 1 redesigned layout with an additional 10 trained HCWs. We assessed the effectiveness of design changes on improving the HCW performance (measured by occurrence and number of risky behaviors) and reducing the physical and cognitive load by comparing the results from phase I and phase III.

**Results:**

The physical load was highest when participants were removing their shoe covers without any balance aid; the use of a chair required the lowest physical effort, followed by horizontal and vertical grab bars. In the revised design (phase III), the overall performance of participants improved. There was a significant decrease in the number of HCW risky behaviors (*P* = .004); 5 risky behaviors were eliminated and 2 others increased. There was a significant decrease in physical load when removing disposable shoe covers (*P* = .04), and participants reported a similar workload in the redesigned doffing layout (*P* = .43).

**Conclusions:**

Through optimizing the design and layout of the doffing space, we reduced risky behaviors of HCWs during doffing of high-level PPE.

## Teaser

The physical design of biocontainment units impacts the contamination risk of healthcare workers (HCWs) during doffing of personal protective equipment. Through optimizing the design and layout of the doffing space, HCWs perform fewer risky behaviors and are safer.

The 2014 Ebola virus disease (EVD) outbreak highlighted the challenge of ensuring the safety of healthcare workers (HCWs) during and after delivery of care to patients with suspected or confirmed serious communicable diseases [1]. When HCWs are potentially fatigued after hours of providing patient care, removal of personal protective equipment (PPE) has been identified as a high-risk activity for self-contamination and potential acquisition of these pathogens [2–8]. Current Centers for Disease Control and Prevention guidelines provide guidance for PPE element selection and doffing procedures while managing people with suspected or confirmed EVD [9]. However, the data to guide the design of the doffing area with regards to HCW safety are limited [10]. Emerging research suggests that environmental design of the biocontainment unit (BCU) impacts the contamination risk of HCWs during doffing [10–13]. We previously found that the layout of the doffing area increases the risk of contamination. For example, items were frequently moved and placed in inconsistent locations when design did not provide standard, convenient locations, leading to errors and risky behaviors; use of different balance aids by HCWs when removing shoe covers had variable success [11, 12, 14–16]. We identified ways that the BCU design can support or disrupt safe doffing and developed a framework of proposed design strategies to promote desirable and safer HCW behavior [14, 16]. By applying some of these strategies, we redesigned the doffing area and then evaluated how design improvements may reduce the HCWs’ physical and cognitive load and the occurrence of risky behaviors that could lead to occupational injury, contamination of the PPE, and contamination of the environment.

**Table 1.** Study design

Phase	Location	N^a^	Balance Aid	Objective	Measures
I	4 BCUs, 1 high- fidelity BCU mockup	38	Chair (n = 10)	To observe HCW behavior in four existing BCUs and a high-fidelity mockup unit and identify key built environment requirements for the doffing area [14]	Performance (Behavioral coding for number and occurrence of risky behaviors) Physical Load (REBA/RULA^b^score) Cognitive Load (NASA TLX^c^ score)
			L-shaped step stool (n = 17)		
			Vertical grab bar (n = 8)		
			No aid (n = 3)		
II A	1 High-fidelity BCU mockup	31	Stool	To compare different balance aids, levels of doffing area demarcation and define the optimized layout	
			L-shaped step stool		
			Vertical grab bar		
			Horizontal grab bar		
II B	1 High-fidelity BCU mockup	9	Vertical grab bar	To compare two optimized layouts and select one to test with HCWs	
			Horizontal grab bar		
III	1 High-fidelity BCU mockup	9	Horizontal grab bar	To observe HCW behavior in the optimized layout and make comparisons with existing BCUs (phase I)	

^a^N represents the number of participants who used a balance aid. In Phase I each HCW used the balance aid used in their unit; in Phase II students did multiple rounds of simulations, using each of the aids provided; in Phase III there was only one balance aid option.

^b^Rapid Entire Body Assessment (REBA) and Rapid Upper Limb Assessment (RULA).

^c^NASA Task Load Index (TLX).

## METHODS

Through a stepwise approach, in 3 phases we assessed how the physical environment can support the high-risk step of removing shoe covers, specifically evaluating 4 stability aids (L-shaped stool, chair, horizontal bar, and vertical grab bar; see Table 1). Our objective was to assess the effectiveness of design improvements of the doffing area on HCW performance, physical load, and cognitive load by comparing the results of phase I (original layout) and phase III (redesigned layout).

In the first phase, we analyzed the BCU layouts at the 4 state-designated Ebola treatment centers in Georgia and observed a series of simulations in all 4 BCUs. We conducted some of the simulations in 1 replicated high-fidelity BCU mock-up that included walls, doors, windows, a bed, and other realistic features built in the SimTigrate Design Lab at the Georgia Institute of Technology. This phase included 41 doffing HCWs (37 nurses, 2 paramedics, 1 physician, and 1 coordinator) and 15 trained observers (TOs), all of whom were trained on using Ebola-level PPE. Participants were asked to don the PPE, perform a simulated patient care task, and then doff the PPE following the step-by-step protocol adopted by their hospital while the TO guided the HCWs through the doffing process.

**Figure 1.**
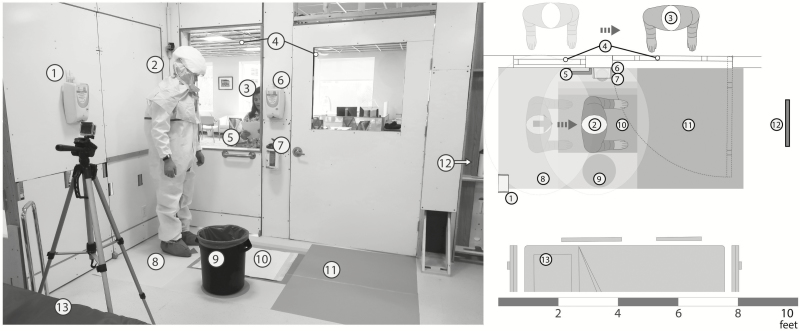
Optimized doffing area design. The healthcare worker (HCW; 2) begins doffing while standing on the arrow in the yellow zone (8), facing the mirror (12), at a 90-degree angle to the trained observer (TO; 3). The HCW uses the horizontal grab bar (5) as a balance aid and uses the mirror to self-inspect when removing disposable shoe covers. After each shoe cover is removed, the HCW steps on the chemical mat in the green zone (10) and disposes of the shoe covers in the trash can (9). The TO dictates the doffing steps and visualizes the HCW using the observation windows (4) that are parallel to the path the HCW is intended to follow while doffing. Primary (6) and backup hand hygiene (1) and wipes (7) are within arm’s reach of the HCW, and the red zone (11) marks the contaminated areas. There is enough clearance between the doffing area and the patient bed (13) for the HCW to avoid stepping in the doffing area while providing patient care.

In the second phase, we tested different balance aids (stool, L-shaped step stool, vertical grab bar, and horizontal grab bar) and different levels of space flexibility with 31 undergraduate students with no PPE training (phase IIA). Based on the framework from phase I and our findings from phase IIA, we designed 2 optimized layouts that we hypothesized would reduce physical and cognitive load and reduce the occurrence and number of observed risky behaviors. We built these layouts in the SimTigrate Design Lab’s high-fidelity BCU mock-up and tested them with an additional 9 students (phase IIB). Using the input from the questionnaire administered to students and results for performance, physical load, and cognitive load, we selected one final layout (Figure 1). In the third phase, we tested the redesigned doffing area layout from phase II in simulations with 10 trained HCWs and 1 TO.

### Performance Assessment

#### Behavioral Coding for Risky Behaviors

Simulations were recorded using between 2 and 5 stationary cameras and an additional handheld camera for phase I and III. We coded each video to identify the number of risky behaviors a HCW performed and tested for and achieved interrater reliability using Pearson 2-tailed correlation (0.89, *P* = .001). We define risky behaviors as actions that could increase the risk of self- and cross-contamination or occupational injury [14, 16]. Based on our previous work, we identified 11 risky behaviors that are impacted by the built environment and defined their risk domain (Table 2) [14–16].

**Table 2.** Observed Risky Behaviors and the Risk They Pose to Healthcare Worker Safety

Risky Behavior	Risk Domain		
	Occupational Injury	Contamination of Personal Protective Equipment	Contamination of Environment
Stretching to reach the balance aid	X		
Sitting while removing disposable shoe covers		X	X
Moving aid in the middle of the task or scooting	X		X
Crossing legs in front of self while removing disposable shoe covers		X	
Not stepping on the chemical mat after removing disposable shoe covers		X	
Having difficulty standing up or adopting an unstable posture	X		
Using hands to push body to stand up or touching the balance aid with both hands			X
Touching the removed disposable shoe cover with both hands		X	
Tossing waste to the trash can or reaching to the trash can	X		X
Missing the opening of the trash can when disposing of items			X
Bumping with the environment	X		X

### Assessment of Physical Load

We measured physical load at the moment of removing the disposable shoe covers using the Rapid Entire Body Assessment (REBA) when participants were standing and the Rapid Upper Limb Assessment (RULA) when participants were sitting. REBA (score range, 1–15) and RULA (score range, 1–13) are assessment tools that measure the physical load of a task and the risk of occupational injury by evaluating posture, with higher scores indicating higher physical load. The score quantifies the position, angle, and twist of upper and lower limbs, the neck, and the trunk with regards to other body parts [17–19].

### Assessment of Cognitive Load

We used the National Aeronautics and Space Administration (NASA) Task Load Index (TLX), a questionnaire tool, to measure the perceived workload of a task on 6 subscales (mental demand, physical demand, temporal demand, frustration, effort, and performance). The TLX scores for perceived workload range from 0 to 100 for all 6 subscales. Higher scores indicate higher perceived workload [20]. Some participants in phase I (n = 19) and all in phase III provided their workload rating on each subscale and after each major task. We focus on the ratings for the shoe cover removal because this task is the one that is most impacted by the built environment and therefore has the most potential to be improved by the new design.

### Statistical Analyses

We analyzed REBA/RULA scores using 1-way analysis of variance to determine if there were differences in physical load when participants used different balance aids. We compared the number of risky behaviors between phase I and phase III using the Mann U Whitney test. We determined the change in occurrence of specific behaviors by calculating the percentage change and tested for the association between phase and the risky behaviors using the Fischer exact test. For phase I, we excluded incomplete data from the analysis and data for 3 HCWs because the TO removed the shoe covers for those HCWs; in phase II data were incomplete for 7 participants; in phase III data were excluded for 1 participant who did not use the balance aid.

Analyses were performed using SPSS Statistics for Windows, version 24.0 (Version 24.0; IBM Corp, Armonk, NY). The Emory University Institutional Review Board approved all research protocols.

## RESULTS

Based on results from phases I and II, we designed an optimized doffing area (Figure 1) by marking the doffing spot that indicates the location to stand during doffing, providing a built-in balance aid within reach of the defined doffing spot, and locating a mirror directly in front of the HCW for self-monitoring and self-inspection. We restricted the location of where the HCW stands and added colored demarcation on the floor to signify the zones and different levels of contamination risk: red, contaminated; yellow, likely contaminated; and green, clean; this is similar to the designations of “hot, warm, and cold” zones [11, 21]. The doffing area has a unidirectional flow from contaminated to cleaner areas to the outside of the patient room in a continuous forward motion. The 2 windows located parallel to the doffing area allow the TO to directly visualize the HCW doffing at all times.

The floor demarcation also indicates the location of key items, such as the trash can and chemical mat, as well as proper location and orientation for the HCW when doffing. We introduced these changes to help reduce the cognitive load of HCWs and prevent items from being moved and placed at inconsistent locations. The zones have various thresholds (indicated by color gradients, item 10 in Figure 1) to accommodate HCWs of different body dimensions with the purpose of reducing the physical load of the HCW and preventing risky behaviors such as bumping with the environment or reaching to the trash can. The size of the doffing area ensures that all items are always within arm’s reach of the HCW (the horizontal grab bar used as a balance aid, primary and backup hand hygiene, and wipes). The trash can and balance aid are located on opposite sides of the chemical mat to encourage the use of one hand to hold on to the bar and the other hand to remove the shoe covers, with the intention of reducing the risk of spreading contamination to the environment. To assist during shoe cover removal, improve posture, and enable self-inspection, we placed a mirror directly in front of the HCW.

### Assessment of Balance Aids

**Figure 2.**
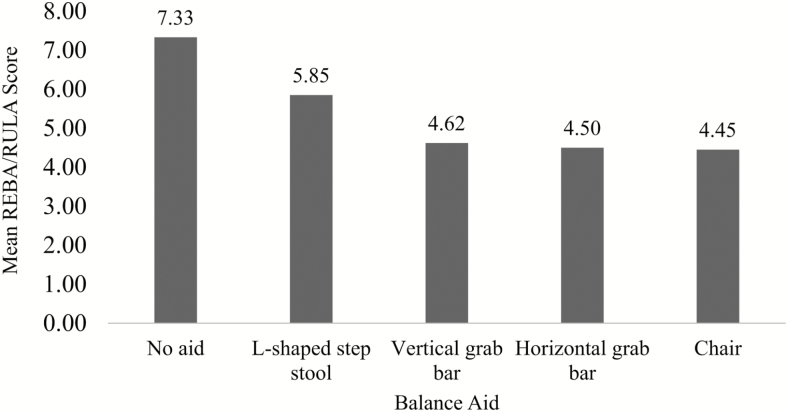
Physical load by balance aid during shoe cover removal. REBA/RULA scores; lower values indicate lower observed physical load. Abbreviation: REBA, Rapid Entire Body Assessment; RULA, Rapid Upper Limb Assessment.

Participants in phase I and III used a chair (n = 10), horizontal grab bar (n = 9), vertical grab bar (n = 8), L-shaped step stool (n = 17), or no tool at all (“no aid”; n = 3) while removing their disposable shoe covers. Participants had the highest physical load when they attempted to remove shoe covers with no aid, while the use of a chair required the lowest physical effort from a HCW (Figure 2). Except for the L-shaped step stool, the physical load was lower when participants used the chair, horizontal grab bar, or vertical grab bar compared with the physical load when they did not use any balance aid (Table 3).

**Table 3.** Mean Difference in Physical Load Using Rapid Entire Body and Upper Limb Assessment When Comparing Balance Aids

	Mean Difference (95% Confidence Interval)				
	No Aid	L-Shaped Step Stool	Chair	Vertical Grab Bar	Horizontal Grab Bar
No Aid	…	1.48 (−0.43, 3.39)	2.88** (0.87, 4.89)	2.71** (0.64, 4.77)	2.83** (0.80, 4.87)
L-Shaped Step Stool	−1.48 (−3.39, 0.43)	…	1.40* (0.19, 2.62)	1.23 (−0.08, 2.54)	1.35* (0.09, 2.61)
Chair	−2.88** (−4.89, −0.88)	−1.40* (−2.62, −0.19)	…	−0.17 (−1.62, 1.27)	−0.05 (−1.45, 1.35)
Vertical Grab Bar	−2.71** (−4.77, −0.64)	−1.23 (−2.54, 0.08)	0.17 (−1.27, 1.62)	…	0.12 (−1.36, 1.61)
Horizontal Grab Bar	−2.83** (−4.87, −0.80)	−1.35* (−2.61, −0.09)	0.05 (−1.35, 1.45)	−0.13 (−1.61, 1.36)	…

*P < .05; ** *P* < .01.

### Changes in HCW Performance

The total number of HCW risky behaviors observed significantly decreased in phase III (median, 1.0) compared with phase I (median, 2.0; *P* = .004). In phase III, we also detected an increase in the percent of HCWs who performed 2 specific risky behaviors: when participants used their hands to push their body to stand up or touch the balance aid with both hands and when participants touched the removed shoe cover with both hands (Table 4). Five other risky behaviors not only decreased but were eliminated in phase III: sitting while removing shoe covers, bumping with the environment, missing the opening of the trash can when disposing of items, tossing waste to the trash can/reaching to the trash can, and moving the (mobile) balance aid in the middle of the task or scooting. There was a significant association between the phase and the observation of HCWs tossing waste to the trash can or reaching to the trash can (*P* = .003). Two of the listed behaviors were not observed in either phase: participants were never seen stretching to reach an aid and never failed to step on the chemical mat.

**Table 4.** Changes in the Occurrence of Risky Behaviors

Risky Behavior	HCWs (N = 38) Who Performed the Risky Behavior in Phase I, n (%)	HCWs (N = 9) Who Performed the Risky Behavior in Phase III, n (%)	Change (%)	Fischer Exact Test *P* Value
Stretching to reach the balance aid	0 (0)	0 (0)	0	…
Sitting while removing disposable shoe covers	3 (8)	0 (0)	−100	1.00
Moving aid in the middle of the task or scooting	6 (16)	0 (0)	−100	.579
Crossing legs in front of self while removing disposable shoe covers	21 (55)	3 (33)	−86	.286
Not stepping on the chemical mat after removing disposable shoe covers	0 (0)	0 (0)	0	…
Having difficulty standing up or adopting an unstable posture	19 (50)	3 (33)	−84	.470
Using hands to push body to stand up or touching the balance aid with both hands	1 (3)	1 (11)	0	.350
Touching the removed disposable shoe cover with both hands	2 (5)	1 (11)	−50	.480
Tossing waste to the trash can or reaching to trash can	21 (55)	0 (0)	−100	.003*
Missing the opening of the trash can when disposing of items	5 (13)	0 (0)	−100	.567
Bumping with the environment	5 (13)	0 (0)	−100	.567

Abbreviation: HCW, healthcare worker.

*P < .05.

### Changes in HCW Physical Load for Shoe Cover Removal

To assess the effectiveness of design improvements on reducing HCW physical load during shoe cover removal, we compared the REBA/RULA scores from phase I (n = 38) and phase III (n = 9; while 10 participants completed phase III, 1 of them did not use the balance aid, therefore, we only report results of 9 participants for this comparison). REBA/RULA scores for phase I (median, 5.5; interquartile range [IQR], 1.5) were significantly higher than those in phase III (median, 4.5; IQR, 1.0; *P* = .04).

### Changes in HCW Cognitive Load for Shoe Cover Removal

**Figure 3.**
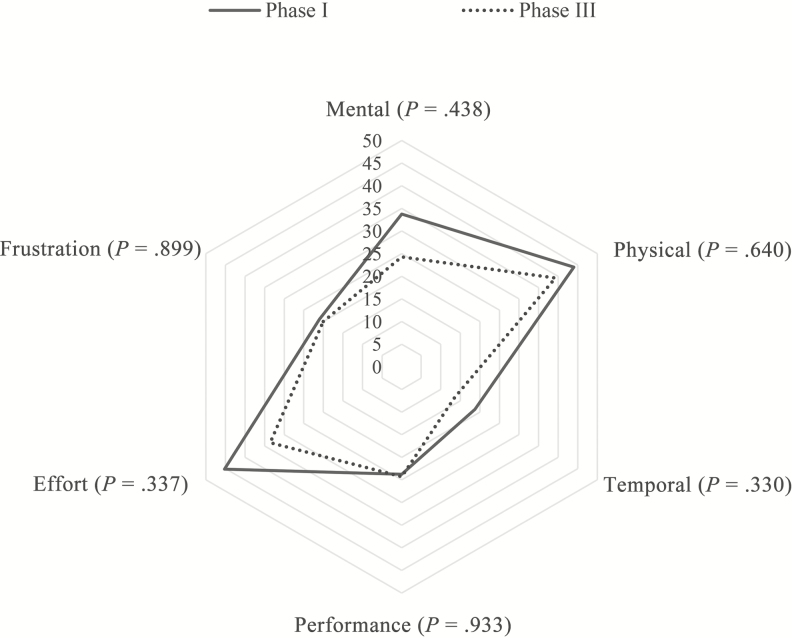
NASA Task Load Index scores for perceived cognitive load during shoe cover removal by subscale. Cognitive load during shoe cover removal for phase I (n = 19) and phase III (n = 9).

To assess changes in HCW cognitive load, we compared the TLX scores from phase I (n = 19) and phase III (n = 9) for shoe cover removal. TLX scores remained similar in phase III (median, 25.0; IQR, 7.5; *P* = .43) as in phase I (median, 27.5; IQR, 19.2). We also compared the task load by subscale using a spider plot (Figure 3) and found no significant differences between scores for phase I and III; although when inspected visually, the scores were lower on some of the subscales.

## DISCUSSION

We demonstrated that the built environment has a measurable impact on HCW contamination risk while doffing PPE after simulating activities associated with the care of patients with a high-consequence pathogen. Through optimizing the design and layout of the doffing space, we were able to make improvements in HCW performance and reduce both the physical and cognitive load of HCWs, thus reducing their risk for self-contamination. The HCWs’ physical load decreased in the optimized doffing environment (phase III) when compared with HCW performance in the initial settings (phase I). The average number of risky behaviors observed per doffing session was reduced, and some risky behaviors were completely eliminated in the optimized doffing setting. While the cognitive load measured using the NASA-TLX for shoe cover removal was not significantly lower, this may be due to the small sample size in phase III. Regardless, the decrease in risky behaviors suggests the importance of design in improving HCW safety in this high-risk environment.

Our redesign involved several strategies including demarcation of the doffing zone to define the location to stand during doffing, providing a built-in balance aid within reach of the defined doffing spot, and locating a mirror directly in front of the HCW for self-monitoring and self-inspection. Several of these strategies have already been implemented at the study sites.

We provided markings on the floor to guide HCW placement during doffing, their orientation, and the location of the chemical mat. By indicating the location of the HCW at every doffing step, we were able to arrange the critical infrastructure (trash can, balance aid, hand hygiene dispenser, wipes) at a more ergonomic position for the HCW to minimize physical effort and reach. Our findings confirm previous research on the usefulness of color-coded zones with clear demarcation and a restricted doffing area [11]. Use of high-contrast coloring in the areas in the optimized doffing layout made it easier for participants to move following a unidirectional flow within the doffing area without having to frequently look at the floor. Providing flexibility in the form of thresholds for the location of the mat allows the HCWs to set up the doffing area so that it is most comfortable and safe for them. Because of the defined space, we eliminated risky behaviors associated with inadequate posture, such as reaching for the trash can, and another based on proximity (tossing waste into the trash can or missing the opening of the trash can). This also likely reduced the cognitive load for the HCW who no longer had to decide where to situate themselves for doffing. We suggest that the balance aid be located to one side of the HCW and the trash can to the other side in order to discourage risky behaviors such as touching the balance aid or the shoe covers with both hands. We recommend that the TO also verbally indicate through the doffing protocol which hand to use for each task and which shoe cover to remove first to further eliminate the need for the HCW to make decisions during this step. This can be decided during training for each particular doffing area and protocol.

We tested 4 balance aids and found that there was a significant difference in the physical load associated with their use. Participants who opted to remove their shoe covers without using any balance aid had the highest physical load, followed by those who used the step stool. Provision of a balance aid is critical to increasing HCW safety during doffing, particularly during shoe cover removal. We suggest using mobile balance aids (such as the L-shaped step stool) only as a last resort for 2 reasons: they will be moved and placed in inconsistent locations, resulting in opportunities for risky behaviors such as stretching to reach the trash can or not stepping on the chemical mat after removing shoe covers, and they are not as stable and sturdy as their built-in counterparts, resulting in physical instability. Corroborating previously reported findings, the lowest physical load was seen for participants who used the chair/stool and vertical grab bar or horizontal grab bar [11]. However, given that sitting increases the risk of spreading contamination to other parts of the PPE, we suggest installing built-in grab bars [11, 14].

In the final design, we provided a fixed horizontal grab bar for balance support, saw a much lower rate of unstable postures, and eliminated the risky behavior of moving the balance aid while in the process of doffing. Grab bars should be placed in a convenient spot because previous studies found that if done improperly, use of a balance aid may increase the contamination risk during shoe cover removal (switching hands or removing shoe covers in the incorrect order) [11].

The design of our optimized doffing space included a mirror placed directly in front of the HCW. The importance of having a mirror for self-inspection was previously reported [11]. While many of the doffing zones included mirrors, they were sometimes placed to the side or back of the HCW or not at eye level [14]. This small adjustment of making the mirror easily visible to the HCW seemed to make a big difference, allowing the HCW to inspect their PPE without having to turn or bend. This is particularly important when the HCW removes the shoe covers, given that the powered air purifying respirator hood limits their ability to see their feet.

There were some limitations to this study. We did not directly measure contamination rates of PPE, HCWs or the environment. However, reducing risky behavior or physical and cognitive load likely reduces contamination risks [15, 16]. We noted an unintended consequence of an increased percentage of HCWs who performed 2 risky behaviors. Our findings were based on observations in 4 BCUs and 1 mock-up and may not be generalizable to all settings, but the general principles still apply. Our sample size was modest in the postintervention testing, which may have led to an underestimation of the impact of our design interventions.

Overall, our findings underscore the importance of the design and layout of the doffing space as a strategy for enhancing HCW safety. When the space and protocol work together, HCW performance can be highly reliable and errors will be rare.

## Notes

***Acknowledgments.*** The authors thank Rachel Dekom for her support in data coding and analysis and Nicholas Pizzolato for his assistance in the construction of the mock-up unit and the coding of observations. The authors thank the participating hospitals and healthcare workers, as well as their administrative teams, and the members of the Prevention Epicenter of Emory and Atlanta Consortium Hospitals.

***Financial support.*** This work was supported by the Centers for Disease Control and Prevention–Prevention Epicenters Program (grant U54CK000164). Emory University Hospital’s participation was supported in part by the National Center for Advancing Translational Sciences of the National Institutes of Health (grant UL1TR000454 from the Atlanta Clinical and Translational Science Institute).

***Supplement sponsorship. This article appears as part of the supplement “Personal Protective Equipment for Preventing Contact Transmission of Pathogens: Innovations from CDC’s Prevention Epicenters Program,” sponsored by the CDC’s Prevention Epicenters Program.***

***Potential conflicts of interest.*** All authors report no potential conflicts. All authors have submitted the ICMJE Form for Disclosure of Potential Conflicts of Interest. Conflicts that the editors consider relevant to the content of the manuscript have been disclosed.

## References

[1] Hewlett AL, Varkey JB, Smith PW, Ribner BS Ebola virus disease: preparedness and infection control lessons learned from two biocontainment units. Curr Opin Infect Dis 2015; 28:343–8. 26098504 10.1097/QCO.0000000000000176 PMC4743738

[2] Casanova LM, Teal LJ, Sickbert-Bennett EE, et al; Centers for Disease Control and Prevention–Prevention Epicenters Program Assessment of self-contamination during removal of personal protective equipment for Ebola patient care. Infect Control Hosp Epidemiol 2016; 37:1156–61. 27477451 10.1017/ice.2016.169

[3] Fischer WA 2nd, Weber D, Wohl DA Personal protective equipment: protecting health care providers in an Ebola outbreak. Clin Ther 2015; 37:2402–10. 26452427 10.1016/j.clinthera.2015.07.007 PMC4661082

[4] Tomas ME, Kundrapu S, Thota P, et al. Contamination of health care personnel during removal of personal protective equipment. JAMA Intern Med 2015; 175:1904–10. 26457544 10.1001/jamainternmed.2015.4535

[5] Guo YP, Li Y, Wong PL Environment and body contamination: a comparison of two different removal methods in three types of personal protective clothing. Am J Infect Control 2014; 42:e39–45. 24679582 10.1016/j.ajic.2013.12.021 PMC7115291

[6] Casanova L, Alfano-Sobsey E, Rutala WA, Weber DJ, Sobsey M Virus transfer from personal protective equipment to healthcare employees’ skin and clothing. Emerg Infect Dis 2008; 14:1291–3. 18680659 10.3201/eid1408.080085 PMC2600382

[7] Zamora JE, Murdoch J, Simchison B, Day AG Contamination: a comparison of 2 personal protective systems. CMAJ 2006; 175:249–54. 16880444 10.1503/cmaj.060094 PMC1513425

[8] Dubost C, Pasquier P, Kearns K, et al. Preparation of an intensive care unit in France for the reception of a confirmed case of Ebola virus infection. Anaesth Crit Care Pain Med 2015; 34:349–55. 26620545 10.1016/j.accpm.2015.10.002 PMC7104235

[9] Centers for Disease Control and Prevention. Guidance on personal protective equipment to be used by healthcare workers during management of patients with Ebola virus disease in U.S. hospitals, including procedures for putting on (donning) and removing (doffing). Ohio Nurses Rev 2014; 89:11–7.

[10] Garibaldi BT, Kelen GD, Brower RG, et al. The creation of a biocontainment unit at a tertiary care hospital. The Johns Hopkins medicine experience. Ann Am Thorac Soc 2016; 13:600–8. 27057583 10.1513/AnnalsATS.201509-587PS

[11] Herlihey TA, Gelmi S, Cafazzo JA, Hall TNT The impact of environmental design on doffing personal protective equipment in a healthcare environment: a formative human factors trial. Infect Control Hosp Epidemiol 2017; 1–6. 10.1017/ice.2017.68 28460655

[12] Herlihey TA, Gelmi S, Flewwelling CJ, et al. Personal protective equipment for infectious disease preparedness: a human factors evaluation. Infect Control Hosp Epidemiol 2016; 37:1022–8. 27291787 10.1017/ice.2016.124

[13] Hallihan GM, Baers JH, Wiley K, et al. Human factors evaluation of simulated Ebola virus disease patient scenarios: system factors associated with donning and doffing during triage, treatment and transport. Calgary, Alberta, Canada: Alberta Health Services, University of Calgary, 2015.

[14] DuBose JR, Matić Z, Sala MFW, et al; Centers for Disease Control and Prevention–Prevention Epicenters Program Design strategies to improve healthcare worker safety in biocontainment units: learning from Ebola preparedness. Infect Control Hosp Epidemiol 2018; 39:961–7. 29909821 10.1017/ice.2018.125

[15] Mumma JM, Durso FT, Ferguson AN, et al; Centers for Disease Control and Prevention–Prevention Epicenters Program, Division of Healthcare Quality Promotion Human factors risk analyses of a doffing protocol for Ebola-level personal protective equipment: mapping errors to contamination. Clin Infect Dis 2018; 66:950–8. 29471368 10.1093/cid/cix957 PMC6927873

[16] Zimring CM, Matić Z, Wong Sala MF, et al; Centers for Disease Control and Prevention–Prevention Epicenters Program Making the invisible visible: why does design matter for safe doffing of personal protection equipment? Infect Control Hosp Epidemiol 2018; 39:1375–7. 30277187 10.1017/ice.2018.206

[17] Sue H, Lynn M Rapid entire body assessment (REBA). Appl Ergon 2000; 31:201–5. 10711982 10.1016/s0003-6870(99)00039-3

[18] Al Madani D, Dababneh A Rapid entire body assessment: a literature review. Am J Eng Appl Sci 2016; 9:107–18.

[19] McAtamney L, Nigel Corlett E RULA: a survey method for the investigation of work-related upper limb disorders. Appl Ergon 1993; 24:91–9. 15676903 10.1016/0003-6870(93)90080-s

[20] Hart SG NASA-Task Load Index (NASA-TLX); 20 years later. In: Proceedings of the Human Factors and Ergonomics Society annual meeting Los Angeles, CA: Sage Publications Sage CA, 2006.

[21] Guttman O, Gardner A Personal protective equipment and simulation: use of chemiluminescent glow sticks as a game changer? Jt Comm J Qual Patient Saf 2015; 41:234–5. 25977251 10.1016/s1553-7250(15)41031-1

